# Pediatric Emergency Cases in the First Year of the COVID-19 Pandemic in a Tertiary-Level Emergency Setting

**DOI:** 10.3389/fped.2022.918286

**Published:** 2022-06-30

**Authors:** Giorgio Cozzi, Idoya Molina Ruiz, Fabiola Giudici, Sara Romano, Veronica Grigoletto, Egidio Barbi, Alessandro Amaddeo

**Affiliations:** ^1^Institute for Maternal and Child Health IRCCS Burlo Garofolo, Trieste, Italy; ^2^University of Trieste, Trieste, Italy; ^3^Unit of Biostatistics, Department of Cardiac, Thoracic, Vascular Sciences and Public Health, University of Padova, Padua, Italy

**Keywords:** children, adolescents, emergencies, emergency department, COVID-19, pandemic

## Abstract

**Aim:**

Emergency cases are uncommon events in the pediatric emergency setting. This study aimed to evaluate the effect of the Coronavirus disease 2019 (COVID-19) pandemic by describing the number and type of pediatric emergency cases that arrived at the pediatric emergency department (PED) of a tertiary-level children’s hospital in Italy.

**Methods:**

We performed a retrospective study, collecting the main features of pediatric emergency patients who arrived during the first year of the COVID-19 pandemic (March 2020–February 2021) compared to the pre-pandemic period (March 2016–February 2020).

**Results:**

During the study period, 112,168 patients were visited at the PED, and 237 (0.21%) were emergency cases, median age of 4 years (IQR: 1–12). In the first year of the pandemic, 42 children were coded as emergency cases compared to 195 (49/year) during the pre-pandemic period. The proportion of emergency cases was stable (0.27% during the COVID-19 period versus 0.20% during the pre-COVID-19 period, *p* = 0.19). No differences were found regarding the age, gender, hour of arrival, and outcome of patients. We found a significant decrease in the proportion of emergency cases related to respiratory diseases (9/42, 21.4% during the COVID-19 period versus 83/195 during the pre-COVID-19 period (42.6%), *p* = 0.01).

**Conclusion:**

In conclusion, our data suggest that the pandemic had a more significant impact on respiratory emergency cases than on pediatric emergencies in general.

## Introduction

The triage process in emergency medicine allows a rapid and precise sorting of children who arrive at the hospital, assigning them to priority levels according to their medical needs.

Children and adolescents in critical conditions, and/or with compromised vital signs, needing immediate medical care or resuscitation maneuvers are assigned as pediatric emergency codes at triage.

Despite a natural variability among departments, pediatric cases triaged with the highest clinical priority code are relatively uncommon (1, 2). At our pediatric emergency department (PED), emergency cases represent less than 1% of visits (2).

Several reports highlighted a delay in the care in the early phase of the pandemic (3–5). Nevertheless, these data were not confirmed in all settings (6, 7). Moreover, most of the reports describing the influence of the Coronavirus disease 2019 (COVID-19) pandemic on PED visits referred only to the first few months of the pandemic (2, 6–8).

This study aimed to investigate the impact of the pandemic on pediatric emergency healthcare utilization at our Institution, comparing the rate and type of emergency cases during the first year of the COVID-19 pandemic to the previous years. Secondarily, we wanted to describe the main features of children and adolescents who arrived at the PED and received an emergency code at triage in the last 5 years.

## Methods

We conducted a retrospective study on the clinical characteristics of children and adolescents who accessed the PED at the tertiary level, university teaching, children’s hospital, Institute for Maternal and Child Health, IRCCS Burlo Garofolo of Trieste, Italy, from March 1, 2016 to February 28, 2021, and who received an emergency code at triage.

Our Institution’s triage process was based on a four-priority level of increasing severity: white for non-urgent cases, green for minor urgency, yellow for urgent, and red for emergencies and resuscitation cases. We collected data about triage code classification for the five periods. Subsequently, we collected each record of patients assigned with an emergency code: the date and hour of the visit, demographical features of the patients, discharge diagnosis, admission status, and ward of destination of admitted subjects.

Discharge diagnoses were grouped into respiratory disease, neurologic disease, injury, psychiatric disease, cardiovascular disease, non-respiratory infections, intoxication, and others. Respiratory diseases included bronchiolitis, viral wheezing, asthma, pneumonia, and croup. Neurologic diseases included status epilepticus, seizures, encephalitis, and ischemic or hemorrhagic stroke. Injuries included amputations, partial amputations, burns, fractures, drawing, head, chest, or abdominal trauma. Psychiatric diseases included severe agitation/aggressiveness, testament, depression, conversion disorder, and anxiety disorder. Cardiovascular diseases included paroxysmal supraventricular tachycardia, heart failure, and cardiac arrest. Non-respiratory infections included urosepsis, sepsis, and gastroenteritis.

### Ethics

The Institutional Review Board (IRB) of the Institute gave ethical approval to the study protocol (RC 10/2020). Due to the retrospective nature of the study, no specific written informed consent was administered.

### Statistical Analysis

Data of enrolled children were summarized by descriptive analysis. Categorical variables were reported through absolute frequencies and percentages.

We divided the study cohort into a COVID-19 group and a pre-COVID-19 group. The pandemic group was defined as patients admitted during the first pandemic year (March 1, 2020–February 29, 2021) and the pre-pandemic group as patients admitted during the same time frames of the four previous years (March 1, 2016–February 28, 2020).

We classified patients by age as follows: neonates (0–28 days of life), infants (29 days–11 months), pre-school children (2–5 years), school children (6–12 years), and adolescents (13–17 years).

The chi-square test and Fisher’s Exact test were used to examine the variables of interest in the differences between the COVID-19 period and the pre-COVID-19 period. Data were entered into an Excel spreadsheet, and statistical analyses were performed using R software (version 4.0.3, 2020). Statistical significance was considered for *p*-values < 0.05 and all tests were 2-tailed.

## Results

During the study period, a total of 112,168 patients were visited at the PED; 96,645 arrived, between March 2016 and February 2020, before the outbreak of the COVID-19 pandemic in Italy, and 15,523 during the first year of the pandemic, from March 2020 to February 2021. Two hundred thirty-seven (0.21%) received an emergency code at the nursing triage evaluation, 195 before the COVID-19 outbreak (pre-COVID-19 group), and 42 during the first year of the pandemic (COVID-19 group). In children classified as emergency cases, the median age was 4 years (interquartile range: 1–12), and 92 (40%) were female.

Table 1 describes the distribution of the emergency codes in the pre-COVID-19 group and COVID-19 group.

**TABLE 1:** Main study results.

Triage priority levels	Pre-COVID-19 group (*N* = 96645)	COVID-19 group (*N* = 15523)	*P* value*
Non-urgent cases	29132 (30.1%)	5266 (33.9%)	<0.001
Minor urgency	60164 (62.3%)	9249 (59.6%)	<0.001
Urgent cases	7154 (7.4%)	966 (6.2%)	<0.001
Emergency cases	195 (0.20%)	42 (0.27%)	0.101
**Emergency cases**	**Pre-COVID-19 group (*N* = 195)**	**COVID-19 group (*N* = 42)**	*P* value*
**Females, *n* (%)**	76 (39.0%)	19 (45.2%)	0.452
**Age, *n* (%)**			
Neonates (0–28 days)	7 (3.6%)	0 (0.0%)	0.213
Infants (29 days–11 months)	26 (13.3%)	4 (9.5%)	0.501
Pre-school children (2–5 years)	82 (42.1%)	23 (54.8%)	0.133
School children (6–12 years)	36 (18.5%)	3 (7.1%)	0.113
Adolescents (13–17 years)	44 (22.4%)	12 (28.6%)	0.406
**Hour of the arrival, *n* (%):**			
8.00–14.00	66 (33.8%)	12 (28.6%)	0.509
14.00–20.00	57 (29.2%)	13 (31.0%)	0.572
20.00–8.00	72 (36.9%)	17 (40.5%)	0.666
**Month, *n* (%):**			
March	26 (13.3%)	4 (9.5%)	0.501
April	10 (5.1%)	2 (4.8%)	0.922
May	13 (6.7%)	3 (7.1%)	0.911
June	13 (6.7%)	7 (16.7%)	0.034
July	10 (5.1%)	6 (14.3%)	0.032
August	11 (5.6%)	2 (4.8%)	0.82
September	16 (8.2%)	2 (4.8%)	0.445
October	19 (9.7%)	4 (9.5%)	0.965
November	14 (7.2%)	4 (9.5%)	0.603
December	24 (12.3%)	4 (9.5%)	0.612
January	19 (9.7%)	4 (9.5%)	0.965
February	20 (10.3%)	0 (0.0%)	0.03
**Discharge diagnosis, *n* (%):**			
Respiratory disease	83 (42.6%)	9 (21.4%)	0.011
Neurologic disease	46 (23.6%)	16 (38.1%)	0.052
Injury	25 (12.8%)	7 (16.7%)	0.508
Psychiatric disease	11 (5.6%)	1 (2.4%)	0.382
Cardiovascular disease	6 (3.1%)	4 (9.5%)	0.059
Non-respiratory infections	5 (2.6%)	0 (0.0%)	0.294
Intoxication	14 (7.2%)	3 (7.1%)	0.993
Other	5 (2.6%)	2 (4.8%)	0.445
**Outcome, *n* (%):**			
Admission to regular ward	114 (58.4%)	22 (52.4%)	0.47
Observation and discharge	79 (40.5%)	16 (38.1%)	0.772
Admission in the ICU	46 (23.6%)	5 (11.9%)	0.095

*According to a Pearson x^2^ test or Fisher Exact test when appropriate.

The rates of non-urgent, minor urgent, and urgent cases dropped (*p* < 0.001, Table 1). Nevertheless, the proportion of emergency cases remained substantially stable compared to the pre-pandemic period (0.27% vs. 0.20%, *p* = 0.103).

Among children classified as emergency cases, no statistically significant differences were found regarding the age, gender, and hour of the arrival of patients between the first year of the pandemic and the years before.

**FIGURE 1:**
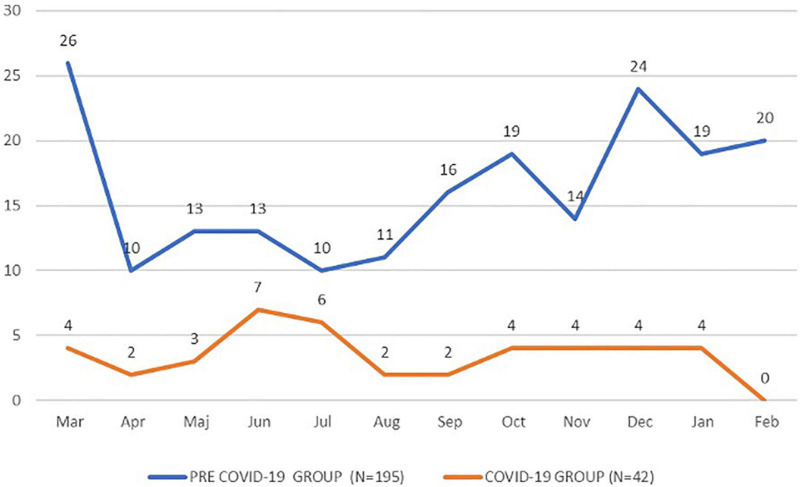
Number of emergency cases per month during the study period.

We noted a significantly different distribution in the month of the arrival of the emergency cases (Figure 1), with a significant drop in February (10.3% vs. 0.0%, *p* = 0.03) and a significant increase in June (6.7% vs. 16.7%, *p* = 0.03) and in July (5.1% vs. 14.3%, *p* = 0.03).

We experienced a statistically significant decrease in respiratory diseases during the first year of the pandemic compared to the pre-pandemic years (42.6% vs. 21.4%, *p* = 0.011).

Among the 237 emergency cases, 137 (57.80%) were admitted to regular wards and 51 (21.51%) to the intensive care unit (ICU). The proportion of patients admitted to the ICU halved during the pandemic year compared to the pre-pandemic period, but this difference was not statistically significant (11.9% vs. 23.6%, *p* = 0.095).

## Discussion

The COVID-19 pandemic and the social measures to counteract its spread have considerably modified the epidemiology of PED cases worldwide (2, 5–11). Considering the first year of the pandemic, this study, performed from a tertiary-level children’s hospital perspective, revealed a drastic decrease in PED visits, 15,523 compared to almost 25/per year before the pandemic, and these data were in line with another report performed in the same period in New York City, United States (12). We described a substantially stable rate of emergency cases, associated with a great decrease in non-emergency cases. These data were similar to the experience in New York City (12). In Italy, COVID-19 began to spread in February 2020. We had a strict national lockdown from March to May 2020. Social distancing measures were suspended during the summer and reimposed from September 2020 until February 2021, without periods of strict lockdown. Previous studies already showed a decrease in the number of PED visits during the pandemic, more pronounced in the periods of strict lockdown (2, 5–8). Nevertheless, they did not focus specifically on pediatric emergency cases, so it was unclear if patients were not seen at the PED for the fear of contagion or because there had less severe diseases. Moreover, some reports highlighted a delay in the care during the very early phase of the pandemic in Italy (4). The value of this study was of focusing specifically on pediatric emergency cases during the entire first year of the pandemic. We did not observe a significant increase in the proportion of emergency cases at our PED, suggesting that there was no significant delay in the care of severe acute diseases, not confirming what was already reported in other settings (5, 13). Notably, we observed a significantly lower proportion of emergency cases related to respiratory diseases and a decreased percentage of children and adolescents needing admission to the ICU. This data was in line with previous studies showing the tremendous impact of the social distancing measures on the spread of respiratory infections (14–16) and suggested a role of these measures in the prevention of severe acute respiratory diseases. During the first year of the pandemic, we did not observe a significant change in the number of other causes of severe acute presentation, such as injuries, intoxication, and psychiatric disorders. Therefore, for these kinds of cases, we did not experience a measurable indirect influence of the social measures imposed to limit the spread of COVID-19. In this study, we described a considerable number of pediatric emergency cases with their main features, and this may be useful for readers interested in the characterization of these uncommon events. Some limitations need to be acknowledged: first, we had to rely on the triage codes assigned by different nurses, therefore, we cannot exclude operator variability. Besides, we focused only on emergencies. Therefore, we were not able to describe the variability of the type of accesses in general. A significant increase in severe PED accesses related to mental health problems was reported (17). Nevertheless commonly used triage tools tend to underestimate the urgency of psychiatric presentations (18). So, we cannot exclude that we have missed some severe psychiatric presentations. Secondly, due to the limited size of the samples, random fluctuations in data cannot be excluded. Third, our data referred to a PED of a single Institution, so the generalizability is limited; however, our hospital is the only pediatric hospital serving the whole area of the Trieste Province (about 230.000 inhabitants) for the pediatric population and this remained unchanged over the 5 years of the study. The triage classification did not change during the study period.

In conclusion, our data showed that during the first year of the COVID-19 pandemic, the proportion of emergency cases at a tertiary-level children’s hospital was stable despite a general reduction of visits to the PED.

Furthermore, we noted a significant decrease in the proportion of severe acute respiratory cases during the first year of the pandemic with a decreased percentage of ICU admissions.

## Data Availability Statement

The raw data supporting the conclusions of this article will be made available by the authors, without undue reservation.

## Ethics Statement

The studies involving human participants were reviewed and approved by Institutional Review Board of the Institute for Maternal and Child Health IRCCS Burlo Garofolo, Trieste, Italy. Written informed consent from the participants’ legal guardian/next of kin was not required to participate in this study in accordance with the national legislation and the institutional requirements.

## Author Contributions

GC and EB designed the study. IM, SR, and VG collected the data. FG performed the statistical analysis and prepared the table. SR and VG drafted the initial version of the study. GC, EB, and AA critically reviewed the manuscript and developed the final version of the manuscript. All authors approved the final version of the manuscript and approved this submission.

## Conflict of Interest

The authors declare that the research was conducted in the absence of any commercial or financial relationships that could be construed as a potential conflict of interest.

## Publisher’s Note

All claims expressed in this article are solely those of the authors and do not necessarily represent those of their affiliated organizations, or those of the publisher, the editors and the reviewers. Any product that may be evaluated in this article, or claim that may be made by its manufacturer, is not guaranteed or endorsed by the publisher.

## Abbreviations

PED: pediatric emergency department.
